# *Angelica gigas* Nakai Has Synergetic Effects on Doxorubicin-Induced Apoptosis

**DOI:** 10.1155/2018/6716547

**Published:** 2018-08-01

**Authors:** Yong-Joon Jeon, Jong-Il Shin, Sol Lee, Yoon Gyeong Lee, Ji Beom Kim, Hak Cheol Kwon, Sung Hun Kim, Inki Kim, Kyungho Lee, Ye Sun Han

**Affiliations:** ^1^Department of Biological Sciences, Konkuk University, Neungdong-ro 120, Gwangjin-gu, Seoul 05029, Republic of Korea; ^2^Korea Institute of Science and Technology, Gangneung, Gangwondo 25451, Republic of Korea; ^3^Department of Convergence Medicine, Asan Institute for Life Sciences, Asan Medical Center, Seoul 05505, Republic of Korea; ^4^Korea Hemp Institute, Konkuk University, Konkuk University, Neungdong-ro 120, Gwangjin-gu, Seoul 05029, Republic of Korea; ^5^Department of Advanced Technology Fusion, Konkuk University, Neungdong-ro 120, Gwangjin-gu, Seoul 05029, Republic of Korea

## Abstract

Natural products are valuable sources for drug discovery because they have a wide variety of useful chemical components and biological properties. A quick reevaluation of the potential therapeutic properties of established natural products was made possible by the recent development of the methodology and improvement in the accuracy of an automated high-throughput screening system. In this study, we screened natural product libraries to detect compounds with anticancer effects using HeLa cells. Of the 420 plant extracts screened, the extract of* Angelica gigas* Nakai (AGN) was the most effective in reducing cell viability of HeLa cells. Markers of apoptosis, such as exposure of phosphatidylserine and cleavage of caspase-7 and PARP, were increased by treatment with the AGN extract. Treatment of the AGN extract increased expression of PKR as well as ATF4 and CHOP, the unfolded protein response genes. In addition, cotreatment of doxorubicin and the AGN extract significantly increased doxorubicin-induced apoptosis in HeLa cells. Decursin and decursinol angelate, which were known to have anticancer effects, were the main components of the AGN extract. These results suggest that the extract of AGN containing, decursin and decursinol angelate, increases doxorubicin susceptibility.

## 1. Introduction

Doxorubicin (adriamycin), belonging to the anthracycline group, was initially derived from* Streptomyces peucetius* in the 1960s [1]. Owing to its wide range of anticancer effects against various types of cancers, including solid tumors and hematological malignancies, doxorubicin has occupied an important place in chemotherapy [2, 3]. The application of doxorubicin, however, has many side effects including cardiac toxicity; therefore, the dose of doxorubicin has been limited [4]. In addition, multidrug resistance or chemoresistance prompted by chemotherapy reduced doxorubicin susceptibility, further limiting its use [5]. Despite these disadvantages, doxorubicin is still an attractive chemotherapeutic drug. To use doxorubicin more efficiently, various therapies have been proposed, including the use of combination therapy as a treatment strategy. Consequently, chemotherapy regimens using doxorubicin, such as FAC (fluorouracil, doxorubicin, and cyclophosphamide), TAC (docetaxel, doxorubicin, and cyclophosphamide), and R-CHOP (rituximab, cyclophosphamide, doxorubicin, vincristine, and prednisone), have been administered to cancer patients [6, 7].

Ever since natural products have been recognized as key components of traditional medicines, many drugs and therapies using natural products have been developed [8, 9]. Consequently, 24 natural products were developed into approved novel drugs between 1970 and 2006 [10]. However, it is very difficult to select the biologically useful natural products from among the wide diversity of natural products. Although high-throughput screening (HTS) is an efficient method for selecting various natural products, it is known to have drawbacks during natural product screening [11]. In this study, we screened natural product libraries via HTS and applied the MTT assay to select the extract of* Angelica gigas* Nakai (AGN), which exhibited anticancer properties.

The genus* Angelica* L. belonging to the Umbelliferae family is distributed in Asia, Europe, and North America and comprises more than 60 species [12, 13]. In China, Japan, and Korea,* Angelica* L. has been used as a traditional herbal medicine for curing colds, pain, and anemia and has been known as the “female ginseng” due to its beneficial effects on female health [12, 14, 15].* Angelica* L. contains a variety of bioactive metabolites, such as pyranocoumarins, essential oils, and polyacetylenes, which exhibit many beneficial effects, including anticancerous, anti-inflammatory, antifungal, antioxidant, and neuroprotective properties [13, 14]. Pyranocoumarins, which are known to be associated with the anticancer effect of* Angelica* L., are more abundant in the roots of the* Angelica gigas* Nakai (local name dang-gui in Korea) growing in Korea than in the species growing in China and Japan [16]. Pyranocoumarins are the major components of the alcoholic extract of AGN [17]. Decursin, decursinol angelate, and decursinol are representative pyranocoumarins in the AGN extract. Decursin, the most abundant pyranocoumarin in the AGN extract, has been reported to show anticancer effects in various cancer cells [13, 14]. In addition, the alleviation of neurotoxicity and nephrotoxicity via its antioxidant properties is the other beneficial effect of decursin [18, 19]. As neurotoxicity and nephrotoxicity are some of the side effects of doxorubicin [20], the combination of doxorubicin and the AGN extract could offer a strategy for increasing the effectiveness of doxorubicin.

Integrated stress response (ISR) is a cellular stress response induced by various stress stimuli, leading to the phosphorylation of eukaryotic translation initiation factor 2 alpha (eIF2*α*). The phosphorylation of eIF2*α* is mediated by four kinases, General Control Nondepressible protein 2 (GCN2), Protein Kinase R (PKR), PKR-like ER localized eIF2*α* Kinase (PERK), and Heme-Regulated Inhibitor kinase (HRI) [21, 22]. Although each kinase recognizes different stimuli, ISR is initiated by very diverse stress stimuli via the four kinases and prompts a cellular response for determining cell fate. The upregulation of eIF2*α* phosphorylation attenuates global protein translation to reduce cellular stress [21]. However, the rates of translation of mRNAs including the second 5′-uORF such as activating transcription factor 4 (ATF4) are further increased by eIF2*α* phosphorylation [23]. ATF4 activates the expression of genes that regulate cellular homeostasis in order to protect cells or increases the expression of downstream transcription factors such as C/EBP-homologous protein (CHOP) [24]. In conditions of severe stress, ATF4 and CHOP induce cell death by activating downstream death factors or by producing ROS via increased protein synthesis [24, 25]. Therefore, ISR is an important mechanism for determining cell fate by inducing a cellular response by various cellular stimuli via the eIF2*α*-ATF4 pathway.

In this study, the AGN extract effectively induced apoptosis in HeLa cells. As the cell death induced by doxorubicin was related to eIF2*α* phosphorylation, we investigated the synergetic effect between doxorubicin and the AGN extract. The administration of the AGN extract enhanced ATF4 and CHOP expression in doxorubicin-treated HeLa cells, resulting in an increase in doxorubicin-induced apoptosis.

## 2. Materials and Methods

Doxorubicin was purchased from Sigma-Aldrich (St. Louis, MO). The PKR inhibitor C16 was purchased from Calbiochem (La Jolla, CA). The anti-phospho eIF2*α*, anti-eIF2*α*, and anti-CHOP antibodies were purchased from Cell Signaling Technology (Beverly, MA). The anti-GAPDH, anti-actin, anti-caspase-3, anti-PKR, anti-Bcl-2, and anti-PARP antibodies were purchased from Santa Cruz Biotechnology (Santa Cruz, CA). The annexin V probe was purchased from Bioacts (Korea). Hoechst 33342 was purchased from Invitrogen (Carlsbad, CA)

### 2.1. Cell Culture

The HeLa cell line was obtained from the Korean Cell Line Bank (KCLB). The HeLa cells were cultured in Dulbecco's modified Eagle's media (Welgene, Daegu, Korea) supplemented with 10% heat-activated fetal bovine serum (Biowest, Nuaillé, France) and 1% penicillin or streptomycin mixtures (GIBCO, ThermoFisher, MA, USA). The HeLa cells were incubated in a humidified atmosphere at a CO_2_ concentration of 5% and a temperature of 37°C.

### 2.2. Cell Viability Assay

The cells were seeded in 48-well plates and incubated for 16 h. The cells were treated with different concentrations of doxorubicin for 24 h. Cell survival was measured using the MTT [3-(4, 5-dimethylthiazol-2-yl)-2, 5-diphenyltetrazolium bromide] assay (Sigma-Aldrich, ST Louis, MO). In brief, PBS containing 5 mg/ml MTT was diluted with the media at a concentration of 0.5 mg/ml and incubated in a humidified chamber containing CO_2_ for 2 h. The medium was aspirated from each well and 200 *μ*l DMSO was added to dissolve the Formazan crystals. The absorbance of each well was measured using a UVM 340 plate reader at a wavelength of 570 nm.

### 2.3. Immunoblot Analyses

The cells were harvested using RIPA lysis buffer [containing 150 mM NaCl, 1% Triton X-100, 1% sodium deoxycholate, 0.1% SDS, 50 mM Tris-HCl, and 2 mM EDTA] along with 1% phosphatase inhibitor and protease inhibitor cocktail (Roche Diagnostics, Germany). The protein concentration was quantified using the Pierce BCA protein assay kit (Thermo Scientific, Australia). Proteins boiled in 1x sample buffer [containing 500 mM Tris-HCl (pH 6.8), 10% SDS, 20% glycerol, 0.05% bromophenol blue, and 1%  *β*-mercaptoethanol] for 5 minutes at 100°C were separated on SDS-polyacrylamide gels. The proteins were electrotransferred to Immobilon-P membranes (Millipore, Temecula, CA) and blotted with the indicated antibodies at 4°C overnight in Tris-Buffered saline containing 0.08% Tween 20 (TBST) and 1% nonfat milk. The membranes were then incubated with horseradish peroxidase-conjugated antibodies at room temperature for 2 h, and the band signal was detected using a LAS-3000 Luminescent Image Analyzer (Fujifilm, Japan). To determine the equal loading of samples, the blots were stripped in stripping buffer [containing 100 mM *β*-mercaptoethanol, 2% SDS, and 62.5 mM Tris-HCl (pH 6.8)] at 50°C for 20 minutes, followed by washing twice with TBST buffer for 15 minutes each time, and reprobed with an antibody specific for *β*-actin or GAPDH.

### 2.4. Measurement of Apoptosis

The cells were cultured in a confocal dish and treated with doxorubicin and/or the AGN extract for 24 h. The cells were washed with PBS and binding buffer (20 mM Hepes at pH 7.4, 150 mM NaCl, and 2.5 mM CaCl_2_). The staining solution was prepared by diluting the annexin V probe and Hoechst 33342 with the binding buffer at concentration ratios of 1:200 and 1:5000, respectively. The cells were stained with the staining solution for 20 minutes. The stained cells were observed using a confocal microscope (Zeiss LSM 800, Carl Zeiss).

### 2.5. Preparation of Crude Extract

The crude extract samples used in this study were provided by Natural Product Library of Korea Institute of Science and Technology, Gangneung Institute, Gangneung, Korea. The natural product library was made from Korean native plants. The preparation of* A. gigas *extract is as follows. The roots of* A. gigas* were purchased in a local oriental medicine market in Bonghwa, Korea, in 2015. The plant materials were authenticated by Professor DS Jang at College of Korean Medicine at Kyung Hee University. The specimen was deposited in KIST Natural Product Library (Deposit number: #BS0622A1). The dried materials (100 g) were cut and extracted twice with 1 L of ethanol by reflux at 60°C for 2 hours. Thereafter, the extract was filtered and concentrated using a rotary evaporator under vacuum at 35°C.

### 2.6. Chemical Composition of the AGN Extract

To investigate the chemical constituents of the AGN extract, LC/MS analyses were performed on an Agilent 1200 HPLC system equipped with UV and ESI-MS detection, using a Phenomenex Luna C18 column (150 × 4.6 mm, 5 *μ*m). The mobile phase used was a linear gradient of 10‐100% acetonitrile in water (containing 0.05% formic acid) for over 30 minutes at a flow rate of 0.7 ml/min. The HPLC chromatogram was monitored at a UV wavelength of 254 nm. Mass analysis was performed using the positive-ion mode. After analyses, the peaks in the HPLC chromatogram were identified by comparing the obtained UV spectra and mass spectra with those of compounds previously reported from* A. gigas*.

### 2.7. Quantitative Real-Time PCR

Quantitative real-time PCR was accomplished with HiPi Real-Time PCR 2× Master Mix SYBR Green (ELPiS Biotechnology, Korea) with 40 cycles. The cycle threshold (cT) was observed in extension step and used for calculation of relative gene expression. Analysis of melting curve was carried out in order to convict specific amplification.

### 2.8. Statistical Analyses

The values in the figures are expressed as the mean ± SD. The figures in this study represent the results of experiments performed more than three times. Statistical analyses of the data obtained from the control and the treated groups were performed by using Student's t-test. Values of P < 0.05 indicate statistical significance.

## 3. Results

### 3.1. Screening of the Most Effective Anticancer Candidate from the KIST Natural Product Library

**Figure 1:**
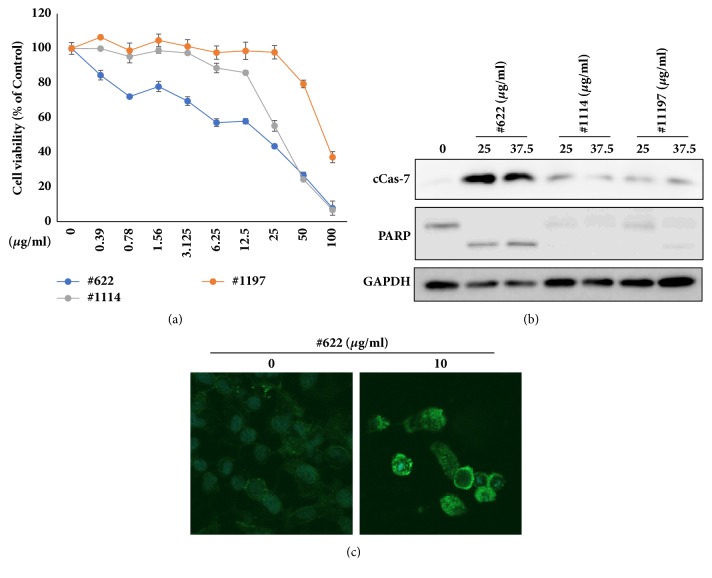
*Angelica gigas* Nakai (#BE0622A1), one of the 420 natural products, showed the most effective anticancer effect. (**a**) The HeLa cells were treated with various concentrations of the indicated natural products for 24 h, and the MTT assay was subsequently performed. (b) The HeLa cells were treated with various concentrations of #BE0622A1, #BE1114A1, and #BE1197A1 for 24 h, and cell lysates were subjected to immunoblot analyses using specific antibodies for cleaved form of caspase-7, PARP, and GAPDH. (c) The HeLa cells were treated with various concentrations of #BE0622A1 for 24 h and apoptosis was analyzed by annexin V staining with FITC conjugation.

Initially, natural product extract libraries were selected to obtain components with anti-inflammatory effects. Since recent studies have shown that cancer and inflammation are closely related [26], in this study we investigated the anticancer effects of natural product extracts from the KIST Natural Product Library. To select extracts with anticancer properties from among approximately 420 natural products, HeLa cells were treated with 50 *μ*g/ml of each natural product extract for 24 hours and natural product extracts that markedly reduced cell viability to below 50% were identified. Through this process, four extracts, #BE0478A1, #BE0622A1, #BE1114A1, and #BE1197A1, were selected. In order to compare the anticancer effects of the four extracts with EC50 value, HeLa cells were treated with various concentrations of each extract for 24 hours and EC50 value of the four natural product extracts was calculated (Table 1 and Figure 1(a)). The results showed that the #BE0622A1 extract was the most efficient in reducing HeLa cell viability, depending on the concentration (Figure 1(a)), and the EC_50_ value was the lowest compared to that of the other extracts (Table 1). Apoptosis has been recognized as an important mechanism for cancer therapy, and many anticancer drugs are known to induce apoptosis in cancer cells [27]. The activation of caspase-7 (cCas-7) and the cleavage of PARP are representative markers of apoptosis. Thus, we compared the apoptosis induced by the extracts by observing cCas-7 activation and PARP cleavage (Figure 1(b)). Similarly to the cell viability, cCas-7 activation and PARP cleavage were enhanced more after treatment with the #BE0622A1 extract than with extracts #BE1114A1 and #BE1197A1.

The apoptosis mediated by the extract #BE0622A1 was also confirmed by annexin V staining (Figure 1(c)). Among the natural products evaluated, #BE0622A1 proved to be the most effective anticancer extract in HeLa cells. The extract #BE0622A1 was prepared from root of* Angelica gigas* Nakai (AGN).

### 3.2. Activation of ISR by Treatment with the Angelica gigas Nakai Extract

**Figure 2:**
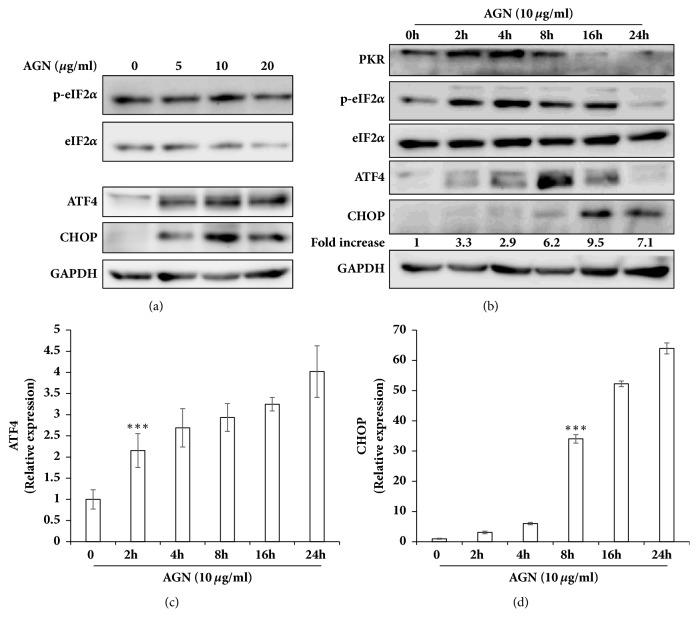
The AGN extract activated the integrated stress response (ISR) in HeLa cells. The HeLa cells were treated with the indicated concentrations of the AGN extract for 16 h (**a**) or with 10 *μ*g/ml of the AGN extract for the indicated time periods (**b**). Cell lysates were subjected to immunoblot analyses using the indicated antibodies. The indicated fold increase in CHOP expression is the ratio of the CHOP to GAPDH. (**c**,** d**) The mRNA levels of ATF4 and CHOP were determined by real-time quantitative PCR. Statistical significance of the difference as calculated by Student's* t*-test is with *∗∗∗*p<0.001.

ISR has been known to cope with diverse stresses, resulting in cell death or adaptation [21]. Four kinases sensing various stress stimuli phosphorylate eIF2*α* to initiate ISR. As the AGN extract is a crude mixture, we speculated that the AGN extract can offer numerous kinds of stimuli to HeLa cells. Accordingly, we investigated whether the AGN extract could increase phosphorylation of eIF2*α*. The EC_50_ value of the AGN extract was approximately 10 *μ*g/ml (Figure 1(a)). Based on these results, the level of eIF2*α* phosphorylation was measured after treatment with the AGN extract for 16 h (Figure 2(a)). The level of eIF2*α* phosphorylation was not affected by concentration of the AGN extract. However, the expression of ATF4 and CHOP, downstream factors of eIF2*α*, was enhanced by treatment with the AGN extract. Phosphorylation of eIF2*α* was increased in a time-dependent manner (Figure 2(b)). The phosphorylation of eIF2*α* was increased after 2 h treatment with the AGN extract. At the same time, the expression of PKR, one of the eIF2*α* kinases, was also increased. The transcription of ATF4 and CHOP was increased in a time-dependent manner, and expression of ATF4 and CHOP was also increased sequentially after 4 h and 8 h, respectively (Figures 2(b)–2(d)). We therefore concluded that the AGN extract-mediated apoptosis was associated with ISR via the eIF2*α*-ATF4-CHOP pathway. Treatment of the AGN extract also increased the splicing of XBP1 mRNA, suggesting activation of the IRE1*α* pathway (data not shown).

### 3.3. The AGN Extract Showed a Synergetic Effect on the Doxorubicin-Induced Apoptosis

**Figure 3:**
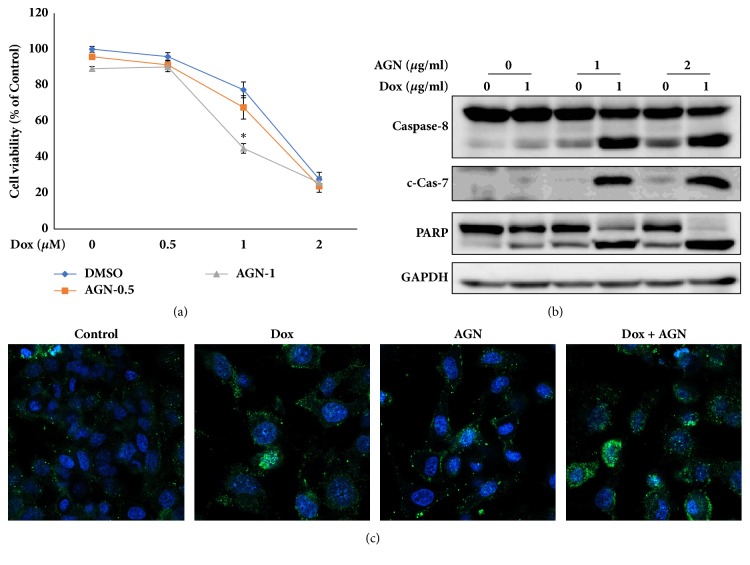
Coadministration of the AGN extract with doxorubicin enhanced doxorubicin-induced apoptosis in HeLa cells. (**a**) The cells were cotreated with the indicated concentrations of doxorubicin and the AGN extract for 24 h, and cell viability was measured using the MTT assay. The statistical significance of the differences, as calculated by Student's t-test, was determined with *∗*p < 0.01. (**b**) The cells were cotreated with the indicated concentrations of doxorubicin and the AGN extract for 24 h and subjected to immunoblot analyses using specific antibodies as indicated. (**c**) The cells were cotreated with 1 *μ*M doxorubicin and 1 *μ*g/ml AGN extract for 16 h and apoptosis was analyzed by costaining with annexin V-FITC and Hoechst probes.

Previous studies have demonstrated that phosphorylation of eIF2*α* improved doxorubicin-mediated cell death in cancer cells [28, 29]. In addition, combination therapy has been widely used as a method for overcoming the limitations of chemotherapy in the treatment of cancer [30]. Therefore, we hypothesized that doxorubicin and the AGN extract could exhibit a synergetic effect based on eIF2*α* phosphorylation. To investigate whether AGN affects doxorubicin-induced cell death, doxorubicin was coadministered with the AGN extract for 24 h (Figure 3(a)). Although cotreatment with 0.5 *μ*g/ml AGN extract and various concentration of doxorubicin hardly affected doxorubicin-induced cell viability, cotreatment with 1 *μ*g/ml AGN extract and 1 *μ*M doxorubicin significantly reduced the cell viability. We then examined the effect of the AGN extract on doxorubicin-induced apoptosis through cCas-7 and PARP. Cotreatment with 1*μ*M doxorubicin with 1 *μ*g/ml or 2 *μ*g/ml AGN extract markedly increased the activation of caspase-7 and the cleaved form of PARP (Figure 3(b)), which means that the doxorubicin-mediated apoptosis was greatly enhanced by the administration of the AGN extract. Furthermore, the administration of the AGN extract along with doxorubicin considerably increased the cleaved form of caspase-8 in contrast to the administration of doxorubicin alone (Figure 3(b)). The upregulation of the cleaved form of caspase-8 indicates that the receptor-mediated/extrinsic apoptotic pathway is activated [31]. The fluorescence intensity of the apoptotic marker as indicated by annexin V staining in the Hoechst-stained cells was also stronger when the cells were cotreated with doxorubicin and the AGN extract than with doxorubicin alone (Figure 3(c)). These results suggest that doxorubicin-induced apoptosis was enhanced via the extrinsic apoptotic pathway by the administration of the AGN extract.

### 3.4. Upregulation of Apoptosis via the ATF4-CHOP Pathway

**Figure 4:**
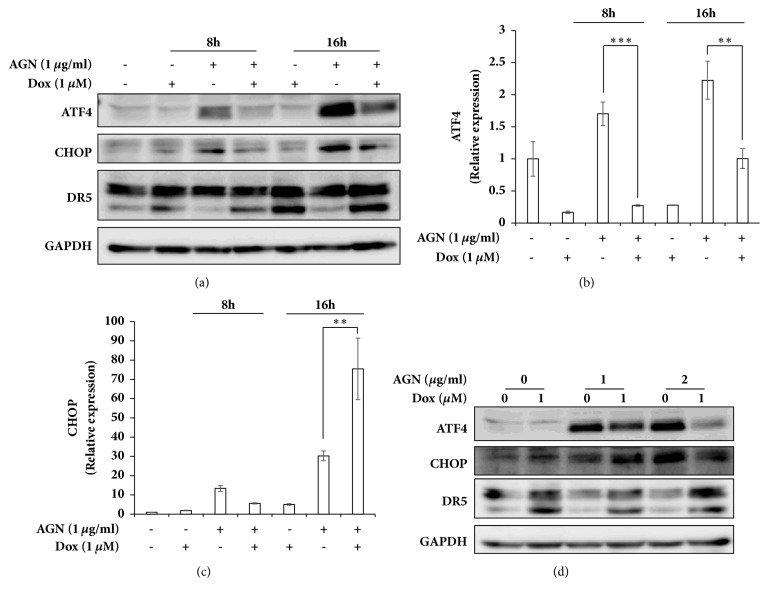
Administration of the AGN extract activated the ATF4-CHOP pathway in doxorubicin-treated HeLa cells. (**a**) The cells were cotreated with 1 *μ*M doxorubicin and 1 *μ*g/ml AGN extract for the indicated time periods and immunoblot analyses were performed using the indicated antibodies. (**b**,** c**) The mRNA level of ATF4 and CHOP were determined by real-time quantitative PCR. Statistical significance of the difference as calculated by Student's* t*-test is with *∗∗*p<0.01 or *∗∗∗*p<0.001. (d) The cells were cotreated with 1 *μ*M doxorubicin and 1-2 *μ*g/ml AGN extract for 24 h as indicated and immunoblot analyses were performed using the indicated antibodies.

The abovementioned results demonstrate that the AGN extract has a synergetic effect on doxorubicin-induced apoptosis. We further speculated whether ISR is correlated to the AGN extract-mediated synergy. To confirm the correlation, HeLa cells were cotreated with doxorubicin and the AGN extract (Figure 4). Figures 4(a)–4(c) represent the time-dependent changes in transcriptional and translational level expression of ATF4 and CHOP at the indicated concentrations, and Figure 4(d) depicts the changes at various concentrations of doxorubicin and the AGN extract over 24 hours. Although the treatment with doxorubicin alone hardly enhanced the expression of ATF4 and CHOP, cotreatment with the AGN extract and doxorubicin increased the expression of ATF4 and CHOP. However, in comparison to the administration of the AGN extract alone, the expression level of ATF4 and CHOP was low by cotreatment (Figures 4(a) and 4(d)). The expression of death receptor 5 (DR5), which is a downstream factor of CHOP and related to caspase-8 activation, was not changed in correspondence with the expression of CHOP (Figure 4). The expression of DR5, therefore, was not related to the ATF4-CHOP pathway. Nevertheless, these results suggest that the synergetic effect of the AGN extract is related to the upregulation of ATF4 and CHOP expression.

### 3.5. Chemical Composition of the AGN Extract

**Figure 5:**
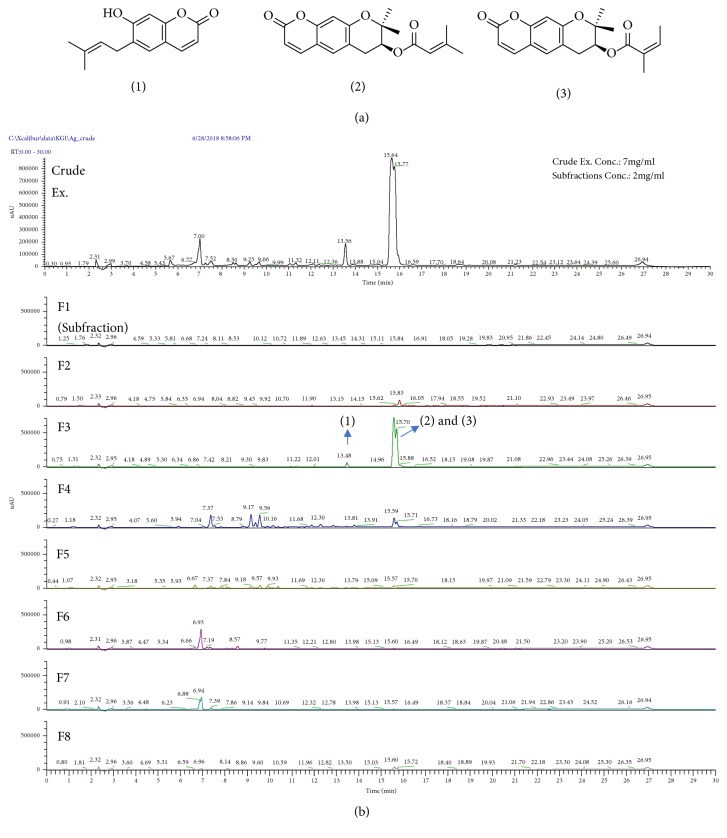
Chemical composition of the AGN extract. (**a**) Chemical structures of the three components: (1) 7-demethylsuberosin, (2) decursin, and (3) decursinol angelate. (**b**) HPLC-MS chromatograms of the AGN extract and the fractions.

Sowndhararajan et al. reported that there are differences in major components depending on the plant part, yield, and extraction method in essential oil of various species of* Angelica* [32]. Therefore, to clarify the major components and biologically active components of the AGN extract, the AGN extract was divided into eight fractions. When HeLa cells were treated with various concentrations of each fraction for 24 hours, fraction #3 reduced HeLa cell viability most effectively (Supplementary Materials, Figure S1a). Also, fraction #3 not only activated caspase 7 but also increased expression of CHOP in HeLa cells (Supplementary Materials, Figure S1b). We then checked the HPLC-MS chromatogram and the 1^H^ NMR spectrum of each fraction to find major components of each fraction.  Figure 5(b) revealed that fraction #3 of the AGN extract contained three coumarins, 7-demethylsuberosin (*m/z* 231), decursin (*m/z* 329), and decursinol angelate (*m/z* 329). Among these substances, decursin and decursinol angelate were the main constituents (Figure 5(b)). These compounds are known as the principal constituents of* A. gigas*, which have significant anticancer effects in various cancer models [8, 9]. Therefore, the results proposed that these compounds play an important role in induction of apoptosis in HeLa cells.

## 4. Discussion

Although doxorubicin is an efficient anticancer drug, various side effects such as drug resistance and cytotoxicity have limited the use of doxorubicin. Many attempts have been made to overcome the limitations of doxorubicin usage and enhance its efficiency, and combination therapy has frequently been used as a strategy for the efficient use of doxorubicin. In the present study, the combination of doxorubicin and the AGN extract markedly enhanced doxorubicin-induced apoptosis in HeLa cells (Figure 3). This event was associated with the AGN extract-mediated expression of ATF4 and CHOP (Figures 2 and 4). Particularly, unlike in HeLa cells, cotreatment of doxorubicin and the AGN extract did not significantly increase doxorubicin-induced apoptosis in wild type WI-38 cells (Supplementary Materials, Figure S2). These results demonstrate that administration of the AGN extract increased the efficiency of doxorubicin in HeLa cells through the activation of ISR, suggesting that the AGN extract works synergistically with doxorubicin.

The AGN extract was the most effective among the natural products screened in inducing apoptosis in HeLa cells (Figure 1). AGN-mediated apoptosis was associated with the eIF2*α*-ATF4-CHOP pathway (Figure 2). Accordingly, we investigated the activity of eIF2*α* kinases PERK and PKR under conditions that the cells were treated with the AGN extract alone. PERK, which is known to be activated by ER stress [21], was not activated by treatment with 10 *μ*g/ml AGN extract (data not shown). On the other hand, the AGN extract increased the expression of PKR, which corresponded to the time when eIF2*α* phosphorylation is increased (Figure 2(b)). Treatment with the PKR inhibitor C16 significantly inhibited the activation of cCas-7 and restored cell viability that had been reduced by treatment with the AGN extract (Supplementary Materials, Figures S3a and S3b). These data show that the AGN extract seems to induce apoptosis by activating PKR in HeLa cells. Treatment with C16, however, neither inhibited nor increased the expression of ATF4 and CHOP (Supplementary Materials, Figure S3c). Previous studies reported that the inhibition of PKR using C16 decreased caspase-3 activation by inhibiting NF-*κ*B-induced inflammation and FADD phosphorylation [33, 34]. p53 is another PKR-mediated apoptotic signal transducer [29, 35]. Therefore, it seems that there is no correlation between the activity of PKR and expression of ATF4-CHOP in AGN extract-treated HeLa cells; upregulation of the eIF2*α*-ATF4-CHOP pathway by treatment with C16 shows the possibility of correlation to another eIF2*α* kinase.

Expression of ATF4 and CHOP was not induced by treatment of doxorubicin alone in HeLa cells, but coadministration with the AGN extract markedly increased the expression of both genes. Indeed, the expression level of ATF4 and CHOP was higher when the cells were treated with the AGN extract alone than with cotreatment (Figure 4). Treatment of breast cancer cells with doxorubicin effectively increases the phosphorylation of eIF2*α* but suppresses the expression of ATF4 at the transcription level [28, 36]. Nevertheless, administration of the AGN extract increased the expression of ATF4 and CHOP in doxorubicin-treated HeLa cells, indicating that ATF4 and CHOP play a role in the synergetic effect of doxorubicin and the AGN extract. Both ATF4 and CHOP are recognized as key transcription factors functioning downstream of eIF2*α*, which regulate the expression of genes associated with cellular homeostasis and cell death [24, 37]. Although many studies indicate that ATF4 plays a role in cellular protection in association with the expression of redox enzymes, autophagy, translation, and multidrug-resistant gene expression [24], ATF4 promotes proapoptotic factors such as Puma and Noxa [38, 39]. Therefore, it is likely that increased expression of ATF4 by the AGN extract enhanced doxorubicin-induced apoptosis in HeLa cells. Indeed, an increase in protein synthesis by ATF4 expression induced ROS-mediated apoptosis [25]. However, treatment with N-acetyl-cystein (NAC), which is a precursor of glutathione, did not affect the cell viability in HeLa cells cotreatment with doxorubicin and the AGN extract (data not shown).

CHOP is a transcription factor functioning downstream of ATF4 and is a well-known death factor in ISR. CHOP-mediated apoptosis is associated with several apoptotic factors including the anti- and proapoptotic Bcl-2 families, microRNAs, TRB3, DR5, and GADD34 [40, 41]. DR5 is known to induce apoptosis by activating caspase-8 [42]. In fact, cotreatment with doxorubicin and the AGN extract significantly increased the activation of caspase-8 (Figure 3). However, administration of the AGN extract did not enhance doxorubicin-mediated DR5 expression (Figure 4). Although the increase in caspase-8 activation by the AGN extract was not related to the expression of CHOP-DR5, the AGN extract is known to activate caspase-8 by enhancing the expression of the DR5 ligand TRAIL [43]. Accordingly, it is possible that upregulation of caspase-8 activation was caused by the expression of TRAIL in AGN extract-treated cells. Inhibition of the anti-apoptotic protein Bcl-2 and activation of the proapoptotic protein Bax/Bak are known as CHOP-mediated apoptotic mechanisms [44, 45]. Treatment with the ANG extract resulted in downregulation of Bcl-2 and upregulation of Bax expression [46]. Also, decursin and decursinol angelate, which are major components of the AGN extract, effectively decreased Bcl-2 expression [43]. Therefore, there is a possibility that CHOP functions as an apoptotic factor in AGN extract-treated cells. However, knockdown of CHOP using specific shRNA did not affect the apoptosis in AGN extract-treated HeLa cells even in cotreated conditions with doxorubicin (Supplementary Materials, Figure S4). Therefore, as mentioned above, CHOP plays an important role in ISR-mediated apoptosis but does not seem to affect AGN extract-mediated apoptosis.

Many studies have shown that decursin and decursinol angelate are major compounds of* Angelica gigas* Nakai, and they are known to have primary responsibility for the anticancer effect of* Angelica gigas* Nakai [13, 14, 46]. Therefore, we also analyzed the composition of the AGN extract and found that decursin and decursinol angelate are major components of the AGN extract (Figure 5). Previous studies have shown that decursin has synergetic effects with doxorubicin [47, 48]. Decursin enhanced caspase-9-mediated apoptosis in doxorubicin-treated multiple myeloma cells, via the mTOR and STAT3 pathways [47] and other reports showed that decursin increased caspase-8-mediated apoptosis by increasing TRAIL sensitivity [49]. Thus, the apoptotic pathway induced by decursin might activate different pathways, depending on the cell's characteristics and conditions. Further studies to characterize the relationship between decursin and doxorubicin are needed. Decursin inhibits the expression of P-glycoprotein, which is an efflux pump that reduces the efficiency of doxorubicin by lowering its cellular concentration [48]. Decursin is also known to inhibit cancer cell metastasis and angiogenesis [50, 51]. Therefore, it could be used as an efficient component in combination therapies along with several other anticancer drugs, including doxorubicin.

## 5. Conclusions

Collectively, our results showed that the AGN extract induced expression of PKR, ATF4, and CHOP as well as phosphorylation of eIF2*α*. It significantly increased apoptosis and enhanced doxorubicin susceptibility in HeLa cells. We also analyzed the composition of the AGN extract and found that decursin and decursinol angelate were the main components of the extract. Consequently, the AGN extract comprising decursin and decursinol angelate could be an effective material for coadministration in combination therapies along with doxorubicin.

## Figures and Tables

**Table 1:** EC50 values of the natural products.

Natural product extract	#478	#622	#1114	#1197
EC 50 value	46.77	10.36	29.12	43.26

## Data Availability

All other data arising from this study are contained within the article and supplementary information files.

## Supplementary Materials

Supplementary Materials 
**Figure S1**: fraction #3 of AGN extract markedly induced apoptosis and increased expression of CHOP in HeLa cells. (a) The MTT assay was performed for measurement of cell viability. (b) The cells were treated with 20 *μ*g/ml fraction of the AGN extract for 16 h. Immunoblot analyses were performed using specific antibodies as indicated.** Figure S2**: AGN extract did not enhance doxorubicin-induced apoptosis in wild type WI-38 cells. (a and b) WI-38 cells were treated with the indicated concentrations of the AGN extract for 24 h. (a) The MTT assay was performed for measurement of cell viability. (b) Immunoblot analyses were performed using specific antibodies as indicated. (c) The cells were cotreated with the indicated concentrations of doxorubicin and the AGN extract for 24 h and cell viability was measured using the MTT assay. (d) The cells were cotreated with 1 *μ*M doxorubicin and 1 *μ*g/ml AGN extract for the indicated time periods and subjected to immunoblot analyses using specific antibodies as indicated.** Figure S3**: C16 restored the AGN-mediated apoptosis regardless of the eIF2*α*-ATF4-CHOP pathway. (a and b) The cells were cotreated with the indicated AGN extract and C16 for 24 h. (a) The MTT assay was performed for measurement of cell viability. Statistical significance of the difference was calculated by Student's t-test with *∗*p<0.01. (b) Immunoblot analyses were performed for measurement of apoptosis using specific antibodies. (c) The cells were cotreated with 10 *μ*g/ml AGN extract and 500 nM C16 for 4 h (top) or 24 h (bottom) and immunoblot analyses were performed using specific antibodies.** Figure S4:** Knockdown of CHOP did not affect the apoptosis in AGN extract-treated HeLa cells. HeLa cells were transfected with EGFP- or CHOP-specific shRNA. (a) The cells were treated with the indicated concentrations of the AGN extract for 24 h, and an MTT assay was performed to determine cell viability. (b) The cells were treated with 10 *μ*g/ml AGN extract for 16 h and subjected to immunoblot analyses using specific antibodies as indicated. (c) The cells were cotreated with the indicated concentrations of doxorubicin and the AGN extract for 24 h and cell viability was measured using the MTT assay. (d) The cells were cotreated with 1 *μ*M doxorubicin and 1 *μ*g/ml AGN extract for the indicated time periods and subjected to immunoblot analyses using specific antibodies as indicated.
