# Supplementary material for: Angelica gigas Nakai Has Synergetic Effects on Doxorubicin-Induced Apoptosis
Source: Biomed Res Int. 2018 Aug 1;2018:6716547. doi: 10.1155/2018/6716547 (PMC6093040; doi:10.1155/2018/6716547)
Supplement: Supplementary Materials — Figure S1: fraction #3 of AGN extract markedly induced apoptosis and increased expression of CHOP in HeLa cells. (a) The MTT assay was performed for measurement of cell viability. (b) The cells were treated with 20 μg/ml fraction of the AGN extract for 16 h. Immunoblot analyses were performed using specific antibodies as indicated. Figure S2: AGN extract did not enhance doxorubicin-induced apoptosis in wild type WI-38 cells. (a and b) WI-38 cells were treated with the indicated concentrations of the AGN extract for 24 h. (a) The MTT assay was performed for measurement of cell viability. (b) Immunoblot analyses were performed using specific antibodies as indicated. (c) The cells were cotreated with the indicated concentrations of doxorubicin and the AGN extract for 24 h and cell viability was measured using the MTT assay. (d) The cells were cotreated with 1 μM doxorubicin and 1 μg/ml AGN extract for the indicated time periods and subjected to immunoblot analyses using specific antibodies as indicated. Figure S3: C16 restored the AGN-mediated apoptosis regardless of the eIF2α-ATF4-CHOP pathway. (a and b) The cells were cotreated with the indicated AGN extract and C16 for 24 h. (a) The MTT assay was performed for measurement of cell viability. Statistical significance of the difference was calculated by Student's t-test with ∗p<0.01. (b) Immunoblot analyses were performed for measurement of apoptosis using specific antibodies. (c) The cells were cotreated with 10 μg/ml AGN extract and 500 nM C16 for 4 h (top) or 24 h (bottom) and immunoblot analyses were performed using specific antibodies. Figure S4: Knockdown of CHOP did not affect the apoptosis in AGN extract-treated HeLa cells. HeLa cells were transfected with EGFP- or CHOP-specific shRNA. (a) The cells were treated with the indicated concentrations of the AGN extract for 24 h, and an MTT assay was performed to determine cell viability. (b) The cells were treated with 10 μg/ml AGN extract for 16 h and subjected to [file 6716547.f1.docx]

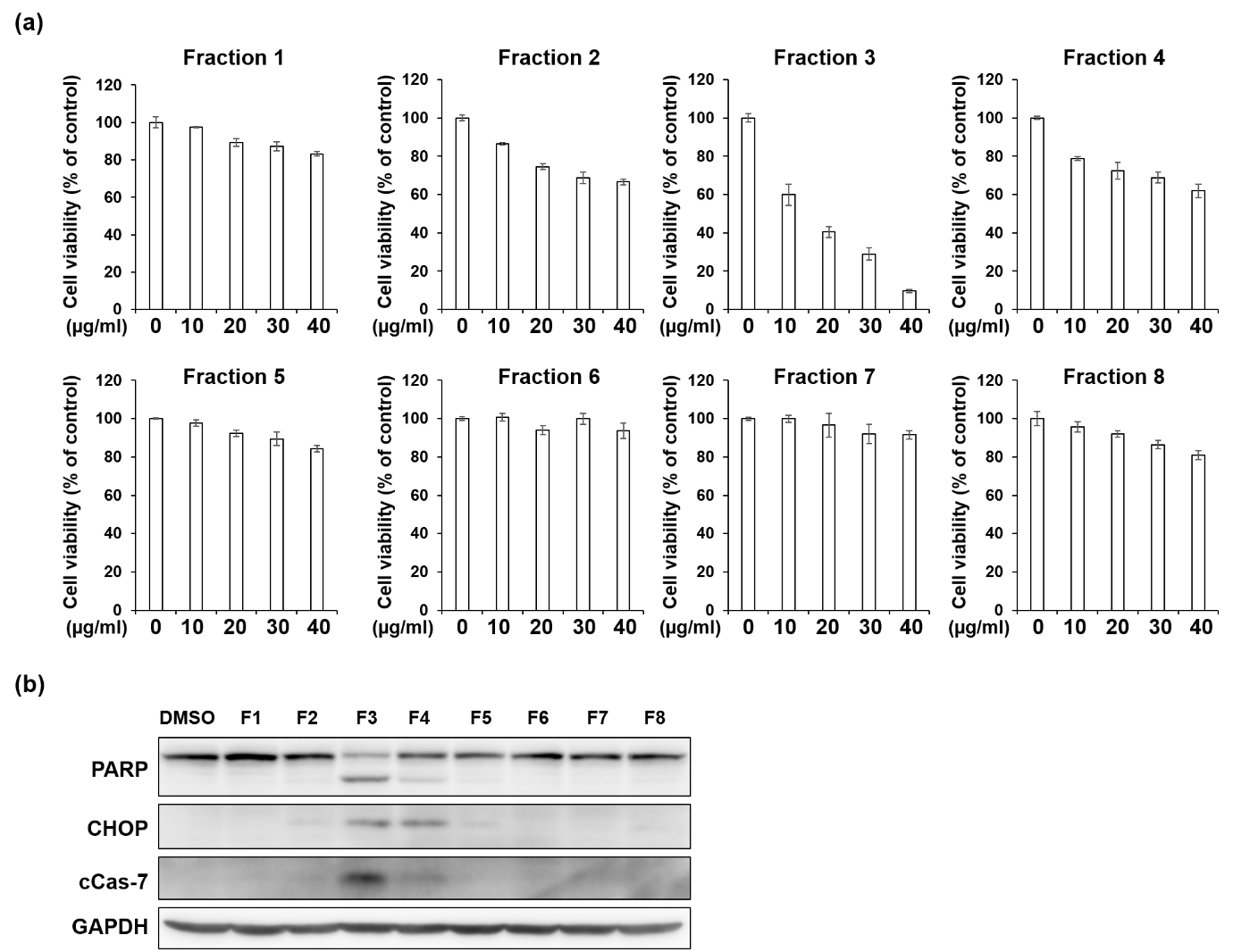


**Figure S1.** The fraction #3 of AGN extract markedly induced apoptosis and increased expression of CHOP in HeLa cells. (a) The MTT assay was performed for measurement of cell viability. (b) The cells were treated with 20 μg/ml fraction of the AGN extract for 16 h. Immunoblot analyses were performed using specific antibodies as indicated.


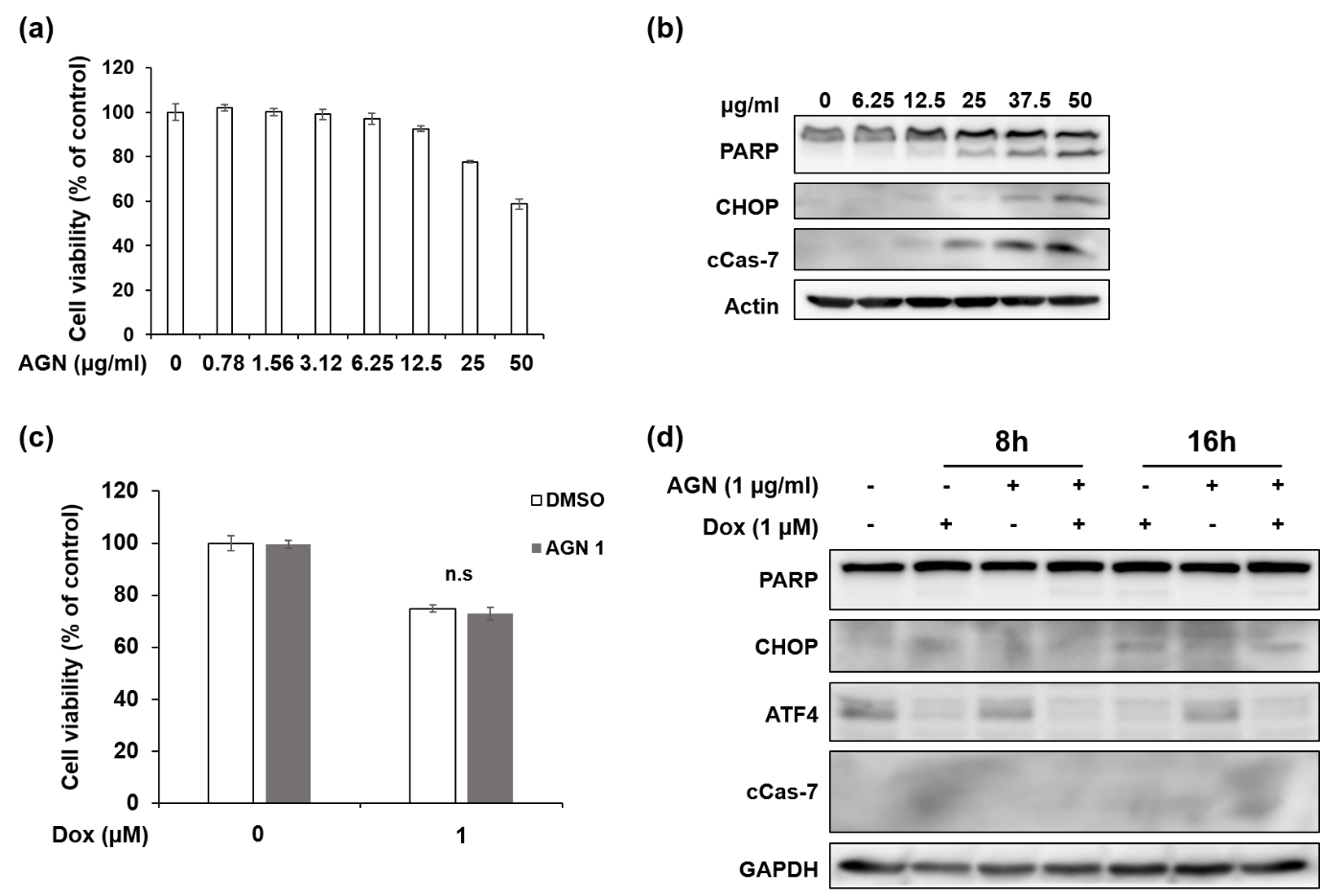


Figure S2. AGN extract did not enhance doxorubicin-induced apoptosis in wild type WI-38 cells. (a and b) WI-38 cells were treated with the indicated concentrations of the AGN extract for 24 h. (a) The MTT assay was performed for measurement of cell viability. (b) Immunoblot analyses were performed using specific antibodies as indicated. (c) The cells were co-treated with the indicated concentrations of doxorubicin and the AGN extract for 24 h and cell viability was measured using the MTT assay. (d) The cells were co-treated with 1 μM doxorubicin and 1 μg/ml AGN extract for the indicated time periods and subjected to immunoblot analyses using specific antibodies as indicated.

**
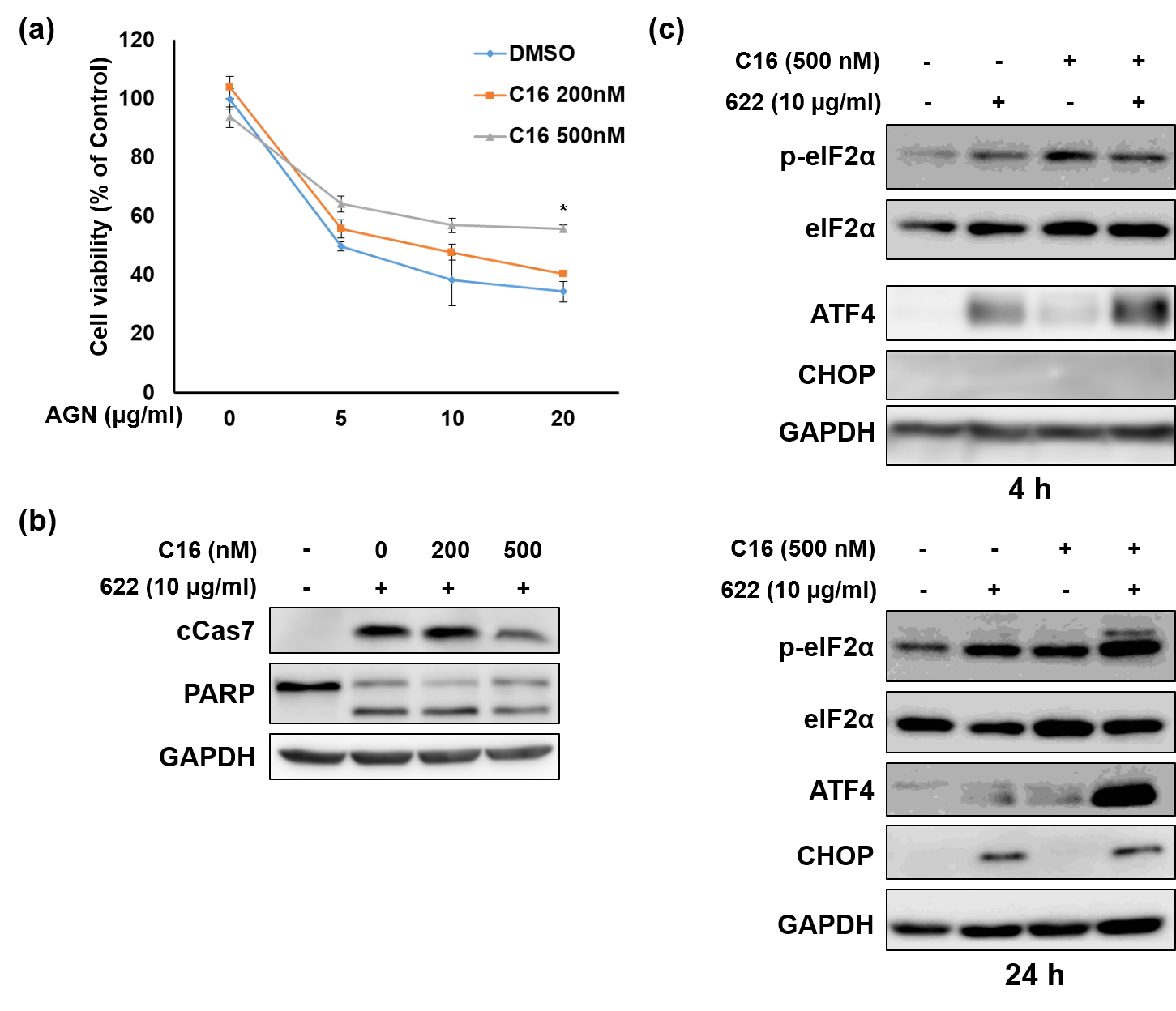
**

**Figure S3**. C16 restored the AGN-mediated apoptosis regardless of the eIF2α-ATF4-CHOP pathway. (a and b) The cells were co-treated with the indicated AGN extract and C16 for 24 h. (a) The MTT assay was performed for measurement of cell viability. Statistical significance of the difference was calculated by the Student’s t-test with *p<0.01. (b) Immunoblot analyses were performed for measurement of apoptosis using specific antibodies. (c) The cells were co-treated with 10 μg/ml AGN extract and 500 nM C16 for 4 h (top) or 24 h (bottom) and immunoblot analyses were performed using specific antibodies.


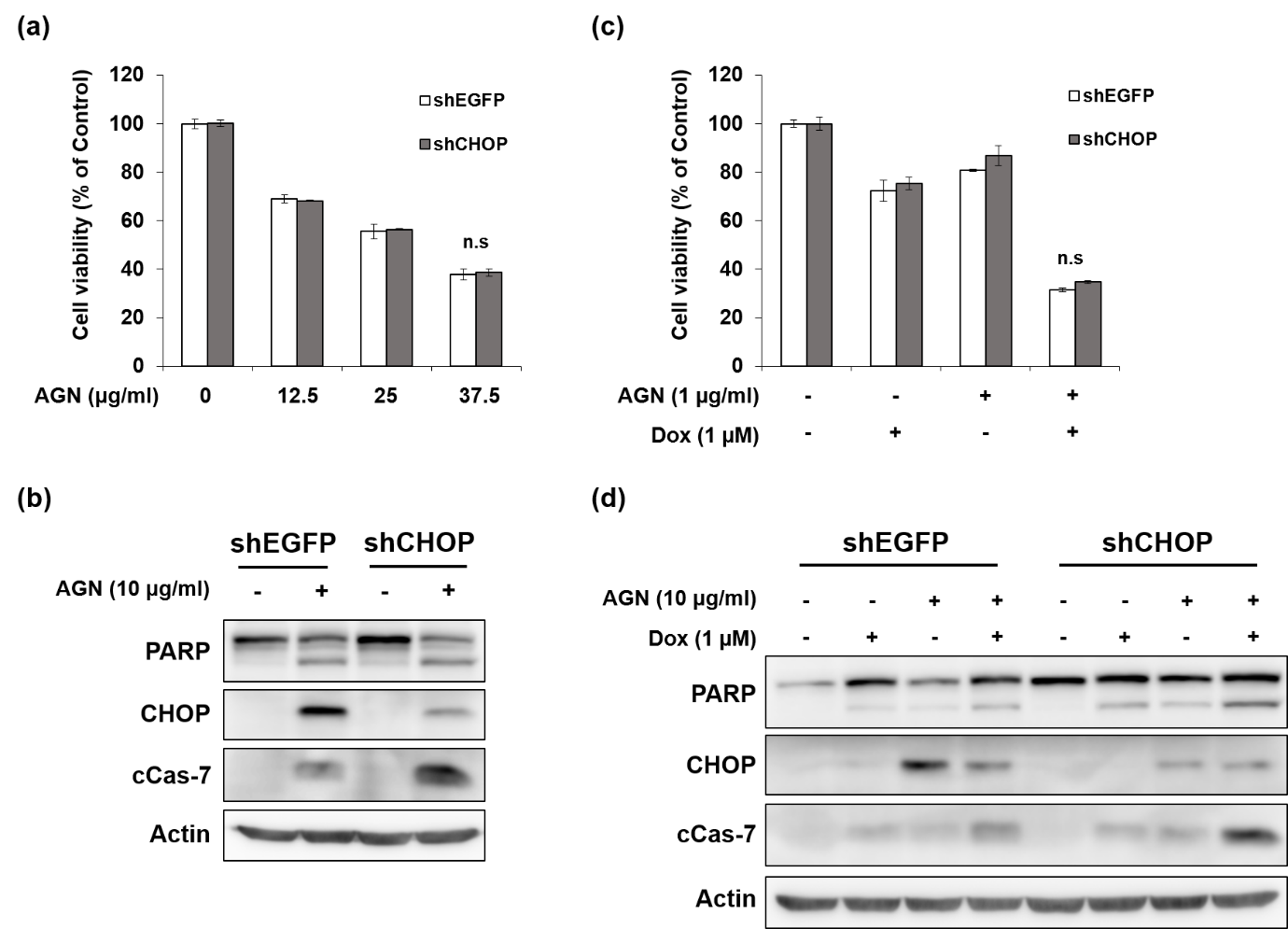


**Figure S4**. Knockdown of CHOP did not affect the apoptosis in AGN extract-treated HeLa cells. HeLa cells were transfected with EGFP- or CHOP-specific shRNA. (a) The cells were treated with the indicated concentrations of the AGN extract for 24 h and an MTT assay was performed to determine cell viability. (b) The cells were treated with 10 μg/ml AGN extract for 16 h and subjected to immunoblot analyses using specific antibodies as indicated. (c) The cells were co-treated with the indicated concentrations of doxorubicin and the AGN extract for 24 h and cell viability was measured using the MTT assay. (d) The cells were co-treated with 1 μM doxorubicin and 1 μg/ml AGN extract for the indicated time periods and subjected to immunoblot analyses using specific antibodies as indicated.
